# CBCT Verification of SRT for Patients With Brain Metastases

**DOI:** 10.3389/fonc.2021.745140

**Published:** 2022-01-19

**Authors:** Judit Papp, Mihály Simon, Emese Csiki, Árpád Kovács

**Affiliations:** ^1^ Department of Oncoradiology, Faculty of Medicine, University of Debrecen, Debrecen, Hungary; ^2^ Doctoral School of Health Sciences, Faculty of Health Sciences, University of Pécs, Pécs, Hungary

**Keywords:** brain metastasis, SRT, HR 3D CBCT, volumetric verification, image guidance

## Abstract

**Background:**

The aim of our work is to demonstrate the role of image guidance and volumetric imaging in stereotactic radiotherapy (SRT) of brain metastases.

**Methods:**

Between 2018 and 2020, 106 patients underwent intracranial stereotactic radiotherapy. 10 patients with metastatic brain tumors treated with SRT were randomly selected and included in our study model. Patients were scanned pre- and post-treatment with cone beam CT. Total of 100 verifications of 50 stereotaxic treatments were performed and analyzed.

**Results:**

Population mean X, Y, Z values were -0.13 cm, -0.04 cm, -0.03 cm, respectively, rotation values 0.81°, 0.51°, 0.46°, respectively. Systematic error components for translational displacements pre corrections were as follows: 0.14 cm for X, 0.13 cm for Y and 0.1 cm for Z. Systematic error components of the post-treatment HR 3D CBCTs were as follows: 0.01 cm for X, 0.06 cm for Y and 0.04 cm for Z.

**Conclusions:**

Population mean values close to 0 confirmed that there is no systematic variation in our system and the accuracy of our equipment and tools is reliable. HR 3D CBCT scans performed pre SRTs further refine patient and target volume setting, support medical decision making and eliminate the possibility of gross error.

## Introduction

Brain metastases (BM) are considered a serious problem regarding the nature of oncological diseases, as they develop in 20-40% of cancer patients during the disease history. BMs are the most common adult brain tumors, with an incidence in Hungary by origin of: lung 40%, skin (melanoma) 30%, breast 25%, gastrointestinal and renal 5-10%. Radiotherapy, either alone or after surgery, remains the mainstay of treatment for brain metastases. Whole brain radiotherapy (WBRT), stereotactic radiosurgery (SRS) and stereotactic radiotherapy (SRT) could be an option (1). Current guidelines are shifting the treatment preferences from WBRT towards stereotactic solutions (SRS, SRT) in cases with a limited number of metastases. These patient`s life expectancy not solely depending on the number of metastasis in the brain but also primary tumor control, Karnofsky score, extracranial mets are factors as well. Therefore more aggressive treatments might be more beneficial for patients with controlled diseases and good overall status (2, 3). Gamma knife SRS is a single session, high dose, focused irradiation. It is used for non-infiltrative intracranial tumors smaller than 3 cm. SRT on a dedicated linear accelerator allows larger lesions to be treated in critical areas of the brain (4–7).SRT is a type of external beam radiotherapy that uses special devices/equipment to position and immobilise the patient in order to deliver high fractional doses of radiation to a well-defined clinical target volume. This significantly reduces normal tissue exposure and subsequent side effects close to the target volume, thus improving the quality of life of patients. SRT can be performed with Gamma knife, Cyberknife, tomotherapy and linear accelerator. The delivery of hypofractionated radiotherapy requires the highest possible reliability and accuracy of equipment, devices and staff (8, 9). Modern linear accelerators with integrated image guided radiotherapy (IGRT) solutions such as cone beam computed tomography (CBCT) enabled the extensive use of SRT in the management of BMs. Non-invasive patient positioning approaches like thermoplastic masks are suitable for fractionated stereotactic treatments of the brain (10–12). CBCT imaging allowed the detection of translational and rotational alignment errors. Furthermore, the six-degrees-of-freedom (6-DOF) robotic couch allowed the correction of rotational alignment errors (13). Single isocentre techniques have been developed to reduce number of isocentres, therefore reduce treatment time (14, 15).State-of-the-art linear accelerators ensure increasingly conformal treatments, and have flattening filter free(FFF) function, therefore increased intensity beam reduces treatment time, ensures that SRT treatments can be performed in 15 minutes or less, as well as door-to-door. At the same time, high-resolution, dynamic volumetric imaging together with an integrated positioning and position determining system, as well as a customisable fixation system are, essential for performing SRT to ensure sub-mm accuracy. In addition, non-invasive 4D imaging, continuous soft tissue monitoring without implanted markers, and protocol-driven interventions are also necessary (16).

The aim of our work is to demonstrate the effectiveness of volumetric imaging by analysing CBCT scans per treatment fraction performed according to our image guidance protocol. We investigated the pre-treatment correction components to determine whether our fixation system is capable of achieving the desired high accuracy of immobilization. In addition, we used post-treatment CBCT scans to verify that the intrafractional displacements were also below the expected level.

## Materials and Methods

Our clinic has 2 adapted Elekta linear accelerators (Synergy, Versa HD), which are capable of performing the most advanced methods of radiotherapy such as IGRT, intensity-modulated radiotherapy (IMRT), volumetric modulated arc therapy (VMAT) and SRT. Their functionality serves the needs of hypofractionated radiotherapy, so that SRT or stereotactic body radiotherapy (SBRT) techniques can be used to safely treat skull, head and neck, chest, abdomen and pelvis targets. The two regions most commonly treated with stereotactic radiotherapy are the brain and the lungs. For this analysis, we selected a cohort of patients treated with SRT for BM to investigate the efficacy of a specific image guidance protocol. Considering the hypofractionated dosimetry scheme, it is of paramount importance to accurately (in millimeters) select, the target area with the help of image guidance. For each patient, 5x6 Gy were delivered every other working day, at a total dose of 30 Gy.

### Patients

Between 2018 and 2020, 106 patients were treated with intracranial stereotactic radiotherapy as per indication, which resulted in. 1060 high resolution (HR) 3D CBCT series of images were registered and corrected based on our verification protocol. To verify our image guidance method, we randomly selected 10 patients from this database who had undergone brain SRT. Thus, our representative sample of 50 stereotaxic fractions contains the measurement results of 100 cone beam CT images. Demographic and clinical data of the patients are shown in **Table 1**. Verification of SRTs was always performed according to an on-line protocol: each pre-treatment, a verification image was taken at the treatment position to determine the submillimetre accurate the patient’s position by a submillimetre accuracy for correction. For image verification, a region-specific preset was used, according to our predefined methodology (**Table 1**).

**Table 1:** Patient characteristics.

Characteristics	No./median	Proportion (%)
Sex		
Male	2	20%
Female	8	80%
Age		
Min	24	
Max	84	
Median	55,5	
PTV volumes		
n	18	
Vmin [cm^3^]	1,2	
Vmax [cm^3^]	103,29	
Vmean [cm^3^]	15,69	
Primary site		
Lung	4	40%
Breast	2	20%
Skin (Melanoma)	1	10%
Ovarium	1	10%
Ependymoma	1	10%
Acusticus neurinoma	1	10%
Indication for SRT		
Intact met.	4	40%
Postop. tumor cavity	1	10%
Postop. tumor cavity+ met.	1	10%
Postop. rec.	2	20%
Postop.rec. + met.	2	20%

### Planning CT

The SRT patients were prepared in the CT simulator, using a Philips Brilliance Big Bore device (Philips, The Netherlands) with a special 85 cm aperture. The scans were performed according to protocol, with a slice thickness of 2 mm and oncological settings. In all cases, the immobilization system used was Qfix (QFix, Avondale, PA, USA). Patients were immobilized in the supine position with a carbon fiber head support and a Moldcare water- activated cushion placed under their head to maintain cervical lordosis. After positioning, an open, kevlar-reinforced 2.4 mm thick thermoplastic mask flap with eye and nose perforations was moulded onto the patients with a bite block fixation device. The number of lesions per patient were either 5 (n=1), 3 (n=1), 2 (n=2) or 1 (n=6).

### Treatment Planning, Dose Prescription

All patients were contoured and planned using Pinnacle (Philips, The Netherlands) irradiation planning system version 9.8. Imaging data from MRI scans performed before localization (T2 and Gadolinium contrast agent enhanced. T1 weighted sequences) were registered into the planning CT sequences by rigid transformation. Treatment target volumes and risk organs were defined based on the information from the MRI scans. The GTV was defined as the contrast enhanced region on T1 weighted MRI scan, the CTV is an isotropic extension of the GTV by 2 mm and the PTV is a further 3 mm extension of the CTV. All treatment plan used a single isocentre approach regardless of the number of lesions. In all cases, the dose was 30 Gy delivered in 5 fractions. Mobius 3D (Varian Medical Systems, Palo Alto, CA, USA) was used for the secondary verification of irradiation plans. Geometric verification during day zero was performed using Mobius 3D software.

### Image Guidance

Radiotherapy of all patients was image-guided and performed on an Elekta Versa HD linear accelerator (Elekta Oncology Systems Ltd, Crawley, UK). The equipment has an Agility MLC head, and uses FFF technique, advanced 2D, 3D and 4D real-time imaging. Volumetric imaging is provided by the high resolution cone beam CT system integrated in the accelerator and its software X-ray volume imaging (XVI). The CB CT is a kilovolt (kV) imaging system with a beam perpendicular to the treatment beam, and it is possible to apply filters and collimators depending on body shape and the region treated. 3D volumetric imaging of the XVI device allows visualization of target volumes and critical organ positions without the need for implanted markers. The XVI is suitable for 3D matching/comparison of planning CT and CBCT images acquired in the treatment position on a bone and soft tissue basis. CBCT scans a region in 2-4 minutes, depending on the data collection method, which is done before/after each treatment fraction. A single turn of the gantry is sufficient for acquisition, the scan range is one full arc. Meanwhile, the cone-shaped X-ray beam on the detector captures a series of two-dimensional summation images of the entire target volume. From the summation images, a 3D reconstruction image database is generated using a special algorithm, due to which no information is lost. The image quality of CBCT scans differs from that of conventional CT scans. The main purpose of CBCT images is to determine the position of the patient. Optimized image quality allows correct image registration using planned CT with minimal patient dose (17). The XVI collects volumetric 3D data series and reconstructs them simultaneously. Imaging is performed at low dose, sub-millimeter isotropic resolution in the treatment setting. HexaPod is an unique, fully robotic patient positioning system. The computer-controlled operating table is capable of independent movements in 6 directions of which are a combination of: translation (along x, y and z axes) and rotation (pitch, roll and yaw) of up to ±3°. The patient positioning devices and the reference frame containing an optical marker are fixed. A high-precision ceiling-mounted infrared camera tracks the 6 optical markers on the reference frame in real time. The reference frame can be dedicatedly fixed to the table top, so that the position of the table and the patient can be calculated. The HexaPOD software unit, which is the iGuide, controls the HexaPod and registers the position of the table. During verification imaging XVI, the cone beam CT software, determines the translation and rotation vectors and transmits them to the iGuide software, which moves the HexaPod and the patient fixed to it in the specified values and directions. The performance of the CBCT scans recorded in our image acquisition protocol fully supported our medical decision making, both in terms of gross error exclusion and target volume localization. HR 3D CBCT scans before brain stereotaxy treatments greatly help to verify the patient’s position, accurately adjust the target volume mm for high-dose radiation treatments. The optimal bone-soft tissue contrast and image quality of diagnostic image verification in the kV range allows for more accurate and safer positioning. This reduces the treatment margins for SRTs, resulting in reduced dose to the tissue and risk organs, which also reduces the incidence of region-specific side effects. HR 3D CBCT values obtained after treatment provide information on the extent of intra-fractional displacements, and body position changes due to organ movements/unintended movements during the treatment period.

### Verification

Patient positioning and immobilisation is followed by the registration of the patient’s position. In iGuide, we record the location of the reference frame and the current position of the table along the X, Y and Z axes. Based on this, iGuide generates a relative table position, which the system will use as a starting point during the correction process. The verification of patients treated with SRT for brain met will be performed according to an image guidance protocol we have defined. For this method, we have created a region-specific preset. This preset consists of 2 series of HR 3D CBCT and 1 series of 3D CBCT. All cone beam CTs were performed under identical technical conditions (collimator: S20, 100 kV, 39.8 mAs, filter: F0). The first pre-treatment high-resolution 3D CBCT is taken in the initial table position. This is used to determine the translational and rotational deviations; during this we register the CBCT images taken in the treatment position to the planning reference CT done in the CT simulator. The XVI software determines the required translational and rotational movements and transmits them to iGuide. Based on the values obtained, medical approval is required to perform the correction. Rotation values can be corrected up to 2.9°, and for values above 3° the patient must be repositioned and reclamped. Translational values are corrected to 10 mm, above that the patient needs to be repositioned. Based on the approved correction values, iGuide will guide the HexaPod to the desired coordinates. As this process takes several minutes, a 3D CBCT is taken immediately before the treatment to check for displacements during the registration process, and the scan is designed to exclude gross error. Once accepted, the SRT fraction can be delivered. The daily fractions are designed from 2 coplanar and 3 non-coplanar half-arc (180°). To cast the 3 non-coplanar arcs, it is necessary to rotate the table from 0°, ± 45°, + 90° isocentre. Immediately post-treatment, another HR 3D CBCT is performed to assess the intrafractional displacements.

## Results

Our analysis compared the results of 50 pre-treatment and 50 post-treatment verification HR 3D CBCT measurements in 10 patients.

**Figure 1:**
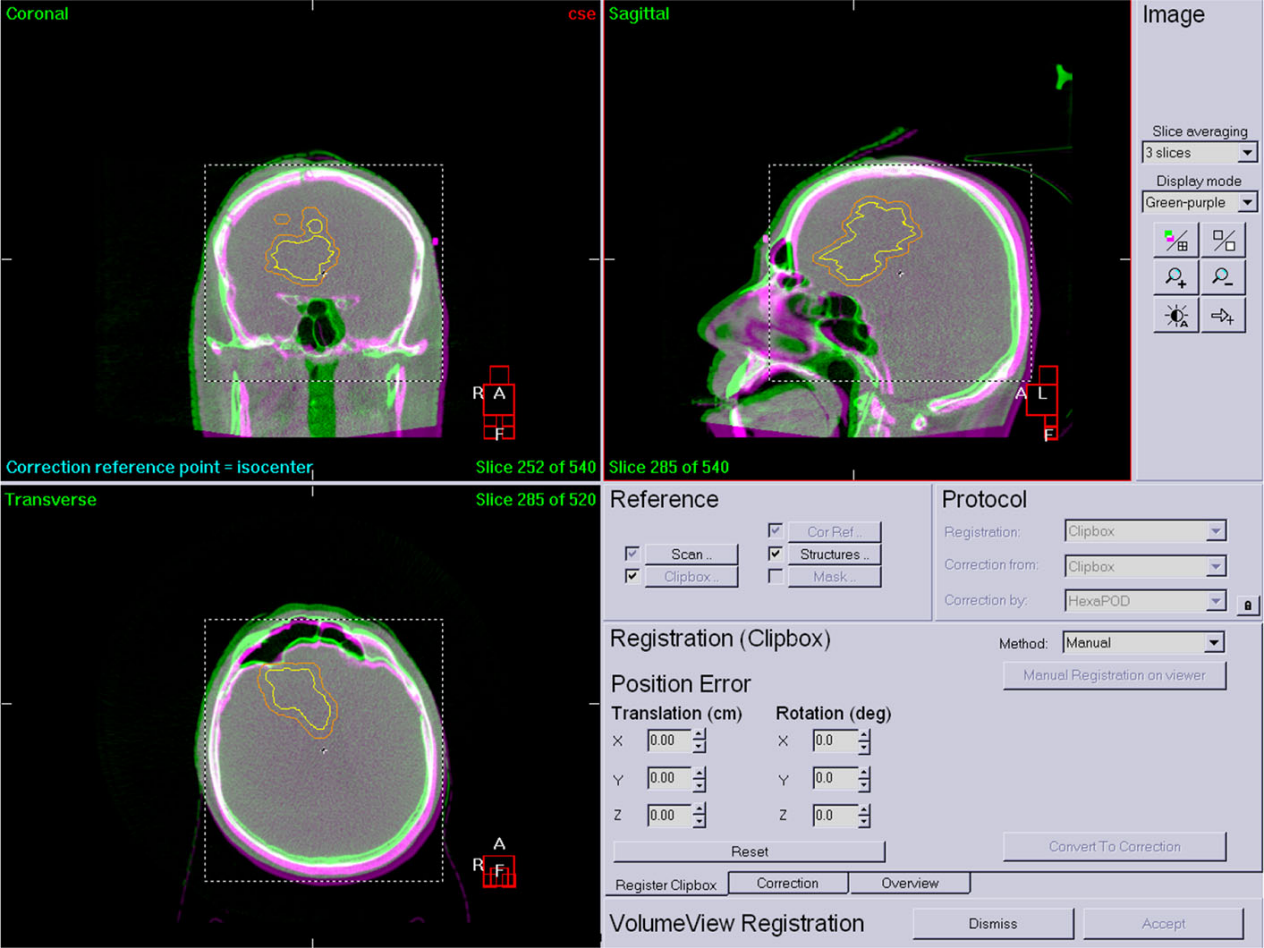
Pre-treatment HR CBCT.

For each patient, 5 fractions were delivered with a fraction dose of 6 Gy on each occasion. All patients’ treatments were complete, with no interrupted SRT. The same bed anchoring system was used in all treatment set-ups (carbon fibre baseplate, Q2 head support plexi, Moldcare mask pad, SRT 2.4 mm mask, bite block, knee support, foot support). All treatments were performed using on-line image-guided patient positioning with HexaPod. **Figure 1** shows a pre-treatment HR CBCT before registration where set-up errors are present (**Figure 1**).

**Figure 2:**
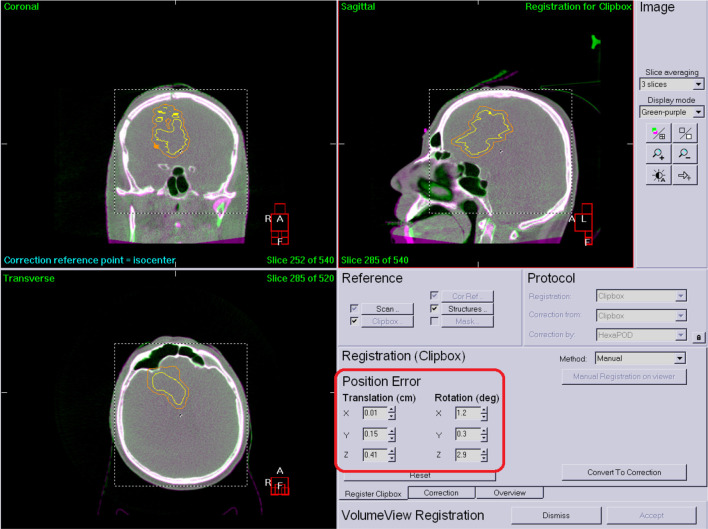
Corrected patient position.

**Figure 2** shows an after registration image. The registration results are highlited with red (**Figure 2**).

**Figure 3:**
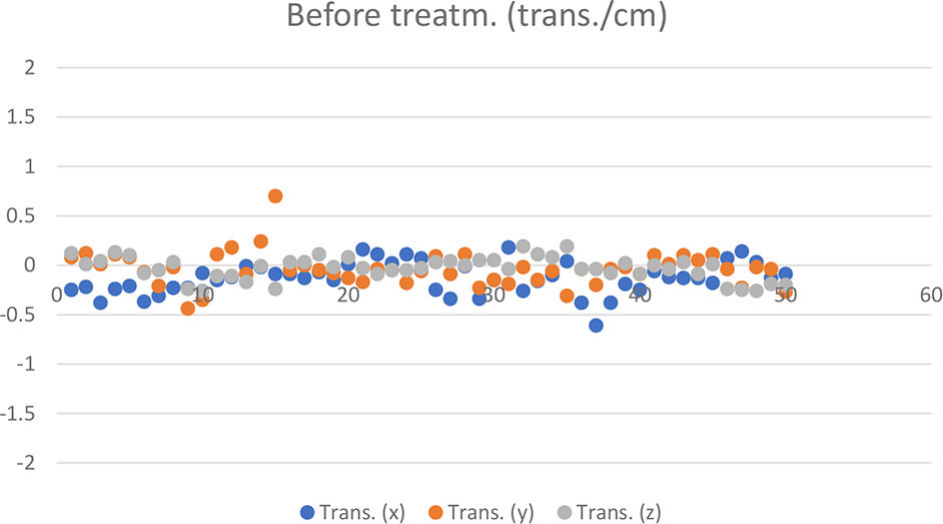
Translational CBCT values recorded in 50 cases before treatment.

**Figure 4:**
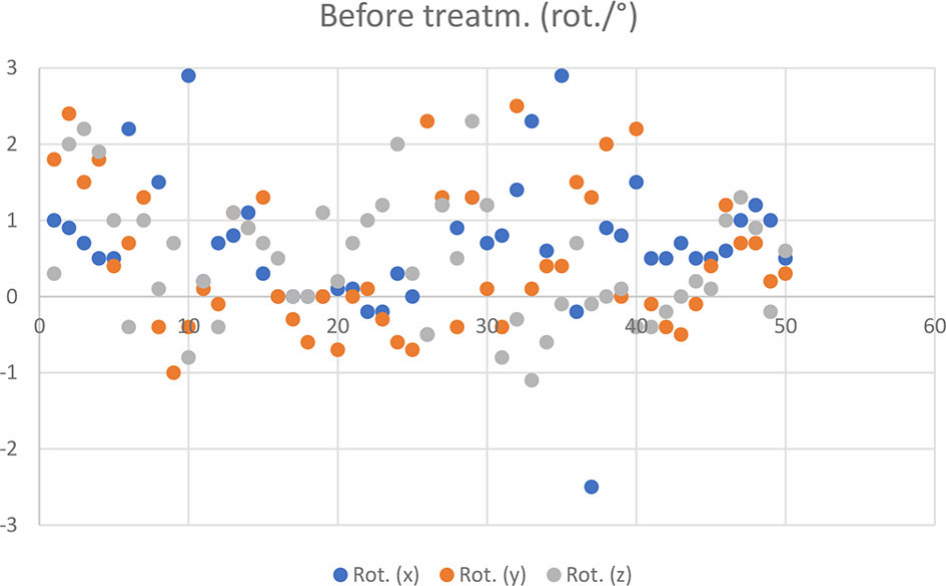
Rotational CBCT values recorded in 50 cases before treatment.

The measurement results of 50 HR 3D CBCTs before treatment are shown in **Figures 3** and **4**.

Based on the registration of HR 3D CBCTs performed during patient set-ups before the treatments, 2 out of 50 fractions required patient repositioning (**Figures 3** and **4**) and re-registration.

On 48 occasions, patients were positioned without gross error using reference markings on the thermoplastic mask.

The mean and standard deviation of the pre-treatment and corrected error components per patient are shown in **Table 2**.

**Table 2:** Mean and standard deviation of pre-treatment CBCTs per patient.

Patient nr.	M/SD	Before treatment					
		Trans. (x) [cm]	Trans. (y) [cm]	Trans. (z) [cm]	Rot. (x) [°]	Rot. (y) [°]	Rot. (z) [°]
1	M	-0,26	0,08	0,08	0,72	1,58	1,48
	SD	0,069	0,043	0,052	0,228	0,736	0,804
2	M	-0,244	-0,218	-0,12	2,88	0,04	0,12
	SD	0,109	0,179	0,125	1,064	0,934	0,746
3	M	-0,078	0,228	-0,128	0,62	0,66	0,5
	SD	0,061	0,292	0,085	0,370	0,623	0,604
4	M	-0,086	-0,062	0,046	0,02	-0,32	0,36
	SD	0,062	0,048	0,050	0,045	0,327	0,462
5	M	0,094	-0,1	-0,05	0,00	-0,30	1,04
	SD	0,052	0,069	0,024	0,212	0,354	0,635
6	M	-0,218	-0,054	0,034	0,72	0,92	0,94
	SD	0,140	0,149	0,021	0,722	1,073	1,031
7	M	-0,06	-0,146	0,106	1,6	0,6	-0,58
	SD	0,173	0,114	0,095	0,982	1,111	0,396
8	M	-0,362	-0,078	-0,046	0,1	1,4	0,06
	SD	0,161	0,073	0,043	1,576	0,863	0,404
9	M	-0,124	0,074	-0,018	0,54	-0,14	-0,06
	SD	0,043	0,043	0,048	0,089	0,351	0,241
10	M	0,004	-0,12	-0,228	0,86	0,62	0,72
	SD	0,112	0,120	0,031	0,297	0,396	0,572

Population mean X, Y and Z values derived from translational components (-0.1334 cm, -0.0396 cm, -0.0324 cm - respectively), rotation values (0.806°, 0.506°, 0.458°- respectively).

Systematic error components for translational displacements before corrections: 0.14 cm for X, 0.13 cm for Y and 0.1 cm for Z.

**Figure 5:**
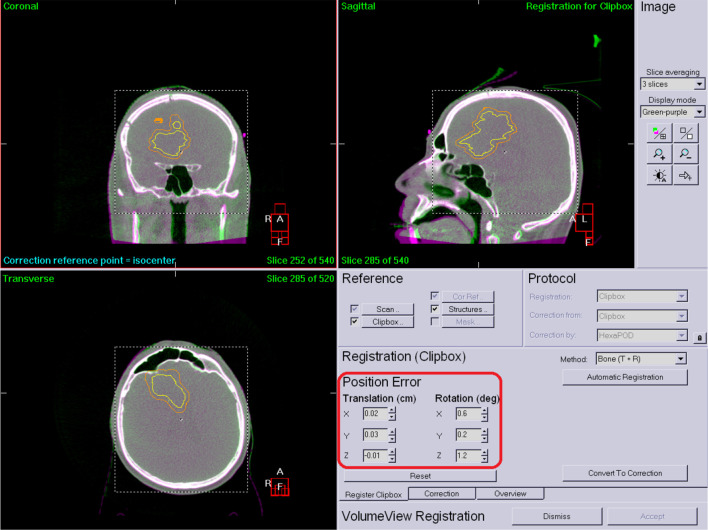
Post-treatment HR CBCT.

**Figure 5** shows the results of a post-treatment HR CBCT, where intrafractional motion would appear (**Figure 5**).

**Figure 6:**
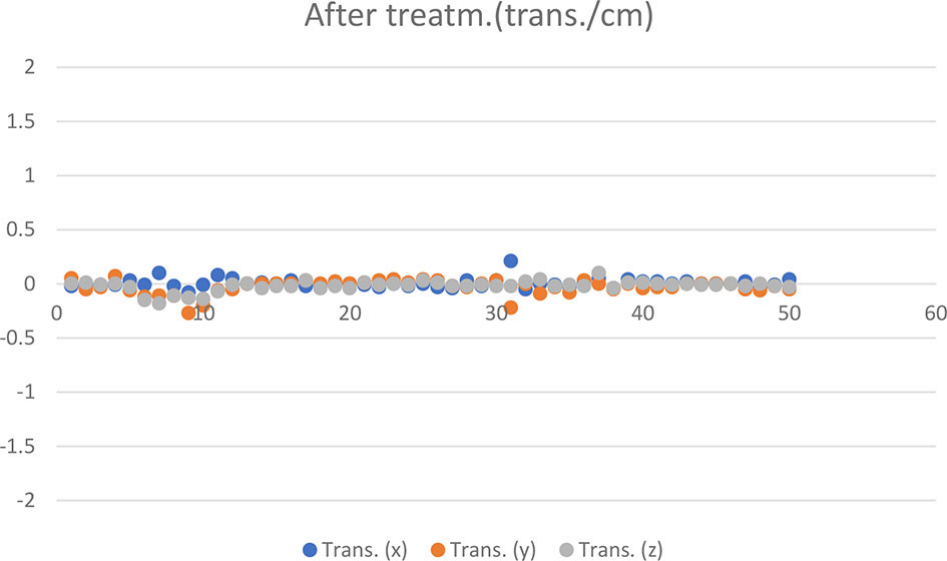
Translational CBCT values recorded in 50 cases post-treatment.

**Figure 7:**
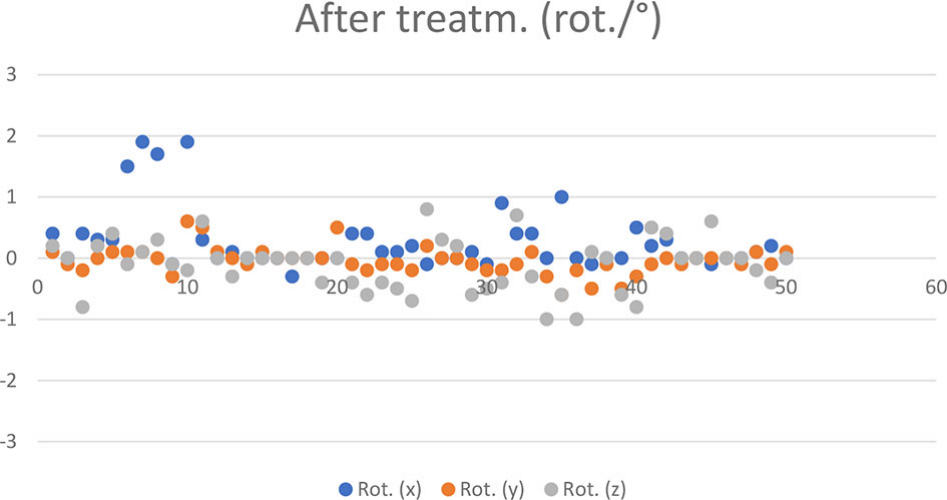
Post-treatment rotational CBCT values recorded in 50 cases.

The mean and standard deviation of the post-treatment and corrected error components per patient are shown in **Table 3**. The post-treatment measurement results of 50 HR 3D CBCTs are shown in **Figures 6** and **7**.

**Table 3:** Mean and standard deviation of post-treatment HR 3D CBCTs per patient.

Patient nr.	M/SD	After treatment					
		Trans. (x) [cm]	Trans. (y) [cm]	Trans. (z) [cm]	Rot. (x) [°]	Rot. (y) [°]	Rot. (z) [°]
1	M	-0,004	-0,004	-0,006	0,28	-0,02	0
	SD	0,019	0,060	0,015	0,164	0,130	0,469
2	M	-0,004	-0,162	-0,142	1,38	0,1	0
	SD	0,065	0,071	0,026	0,844	0,324	0,2
3	M	0,028	-0,022	-0,028	0,08	0,12	0,06
	SD	0,036	0,030	0,028	0,130	0,228	0,329
4	M	-0,004	0,01	-0,018	-0,06	0,1	-0,08
	SD	0,021	0,014	0,029	0,134	0,224	0,179
5	M	-0,01	0,03	0,00	0,24	-0,14	-0,52
	SD	0,013	0,015	0,017	0,152	0,055	0,130
6	M	-0,006	0,002	-0,012	-0,02	-0,02	0,04
	SD	0,034	0,028	0,013	0,084	0,148	0,586
7	M	0,024	-0,084	0,002	0,54	-0,22	-0,32
	SD	0,106	0,084	0,027	0,410	0,259	0,630
8	M	0,018	-0,012	0,012	0,08	-0,32	-0,46
	SD	0,035	0,033	0,054	0,239	0,179	0,488
9	M	0,008	-0,012	-0,006	0,08	-0,04	0,3
	SD	0,011	0,016	0,005	0,164	0,055	0,283
10	M	0,004	-0,036	-0,014	0,06	0	-0,12
	SD	0,027	0,025	0,013	0,089	0,100	0,179

Systematic error components derived from the standard deviation of the mean of the translational components at post-treatments CBCT: 0.01 cm for X, 0.06 cm for Y, and 0.04 cm for Z.

## Discussion

The use of stereotactic treatments such as SRT in the treatment of BMs is increasing in the cohort of patients with few metastases. Although rigid immobilization and long treatment times can lead to patient discomfort and patient movement (18, 19). Frameless stereotactic techniques and treatments had been published previously (19–21) in the literature. Frameless immobilization allows fractionation of the treatments but requires a very high degree of accuracy and reproducibility in patient positioning. In our study we evaluated the patient positioning and interfractional accuracy of our frameless system. Population mean values of each directional displacement components shows that there are no underlying systematic errors remained in our system. The intrafractional displacements can be minimised with the used fixation system, as shown by the results derived from the data measured during post-treatment CBCTs. Measurement results from CBCTs before and after SRTs have demonstrated that our verification protocol and the fixation systems we use are capable of achieving the positioning accuracy required during SRT treatments. Wong et al. (19) reported similar values for mean (0.7 mm) displacement of the isocentre with a stereotactic mask fixation system. Minniti et al. (22) reported 0.08 mm, 0.04 mm and 0.06 mm in cranio caudal, medio-lateral and anterior-posterior intrafractional displacements between CT verification and post-treatment CT respectively. Hamilton et al. (20) reported a mean of 1.8 mm accuracy for a rigid head-mask immobilization system. The advantages of volumetric imaging techniques for the verification of stereotactic radiotherapy are the following: Changes and deviations in the patient’s irradiation position can be accurately tracked and quantified during the treatment. Deviations can be corrected immediately in all directions, along all the axes of rotation. This is of paramount importance for tumors located close to critical organs, such as intracranial tumors, where visualisation of the tumor and its surroundings plays a huge role in medical decision-making (17). Repeated CT verification images bring high resolution datasets and consistency into image analysis (23) Image registration, the HR 3D CBCT technique and the coordinated image guidance system create safe conditions for performing SRTs.

## Conclusions

Our data suggests that correct patient positioning was achieved during the planning CT, which could be successfully reproduced before treatment fractions, without the need for frequent repositioning. The desirable value of the population averages should be close to 0, so that there be no hidden systematic error in the system. In our case, the results obtained show that we have no systematic error during either preparation or execution.

The main limitation of this study is the number of patients, which is a small population and is insufficient for statistical measurements. Further limitation is the retrospective manner of this study which can introduce bias in patient selection and further limits the statistical capabilities of the study.

## Data Availability Statement

The original contributions presented in the study are included in the article/supplementary material. Further inquiries can be directed to the corresponding author.

## Ethics Statement

Ethical review and approval were waived for this study, due to the fact, that patients involved in this study were prepared, planned and treated according to Institutional protocol, no ethical questions could emerge regarding the treatments.

## Author Contributions

Conceptualisation, JP and MS. methodology, JP and EC. investigation, JP and MS. data curation, MS. Writing—original draft preparation, JP. writing—review and editing. JP and ÁK. Supervision, ÁK. All authors have read and agreed to the published version of the manuscript.

## Conflict of Interest

The authors declare that the research was conducted in the absence of any commercial or financial relationships that could be construed as a potential conflict of interest.

## Publisher’s Note

All claims expressed in this article are solely those of the authors and do not necessarily represent those of their affiliated organizations, or those of the publisher, the editors and the reviewers. Any product that may be evaluated in this article, or claim that may be made by its manufacturer, is not guaranteed or endorsed by the publisher.
